# Automatic estimation of knee effusion from limited MRI data

**DOI:** 10.1038/s41598-022-07092-9

**Published:** 2022-02-24

**Authors:** Sandhya Raman, Garry E. Gold, Matthew S. Rosen, Bragi Sveinsson

**Affiliations:** 1 A. A. Martinos Center for Biomedical Imaging, Massachusetts General Hospital, Boston, MA USA; 2 Department of Radiology, Stanford University, Stanford, CA USA; 3 Harvard Medical School, Boston, MA USA; 4 Department of Physics, Harvard University, Cambridge, MA USA

**Keywords:** Biomedical engineering, Magnetic resonance imaging

## Abstract

Knee effusion is a common comorbidity in osteoarthritis. To quantify the amount of effusion, semi quantitative assessment scales have been developed that classify fluid levels on an integer scale from 0 to 3. In this work, we investigated the use of a neural network (NN) that used MRI Osteoarthritis Knee Scores effusion-synovitis (MOAKS-ES) values to distinguish physiologic fluid levels from higher fluid levels in MR images of the knee. We evaluate its effectiveness on low-resolution images to examine its potential in low-field, low-cost MRI. We created a dense NN (dNN) for detecting effusion, defined as a nonzero MOAKS-ES score, from MRI scans. Both the training and performance evaluation of the network were conducted using public radiological data from the Osteoarthritis Initiative (OAI). The model was trained using sagittal turbo-spin-echo (TSE) MR images from 1628 knees. The accuracy was compared to VGG16, a commonly used convolutional classification network. Robustness of the dNN was assessed by adding zero-mean Gaussian noise to the test images with a standard deviation of 5–30% of the maximum test data intensity. Also, inference was performed on a test data set of 163 knees, which includes a smaller test set of 36 knees that was also assessed by a musculoskeletal radiologist and the performance of the dNN and the radiologist compared. For the larger test data set, the dNN performed with an average accuracy of 62%. In addition, the network proved robust to noise, classifying the noisy images with minimal degradation to accuracy. When given MRI scans with 5% Gaussian noise, the network performed similarly, with an average accuracy of 61%. For the smaller 36-knee test data set, assessed both by the dNN and by a radiologist, the network performed better than the radiologist on average. Classifying knee effusion from low-resolution images with a similar accuracy as a human radiologist using neural networks is feasible, suggesting automatic assessment of images from low-cost, low-field scanners as a potentially useful assessment tool.

## Introduction

Osteoarthritis (OA) is a debilitating joint disease, estimated to affect 27 million adults in the United States^1^ and leading to medical care expenditures of close to $200 billion annually^2^. While OA used to be thought of primarily as wear and tear of the joint cartilage, it is now considered a disease of the whole joint, affecting cartilage, bone, ligaments, and joint fluid accumulation^3^. OA becomes more prevalent with age, which is the strongest risk factor for the disease^1^. With an aging US population^4^, an increased demand for methods for OA diagnosis can therefore be expected. This includes medical imaging technologies such as magnetic resonance imaging (MRI), which is frequently listed as the preferred cross-sectional imaging technology of choice for a wide variety of indications in the extremities^5^. However, musculoskeletal MR imaging is already experiencing substantial growth, with a 350% increase in use rate in 1996–2005^5^. This leads to increased medical costs from imaging diagnosis, which were estimated as 19% of all Medicare imaging spending in 2006^6^. There is therefore increased urgency for developing more efficient imaging and diagnosis of OA and its related conditions using MRI.

Recently, the development of Artificial Intelligence (AI) and Deep Learning (DL) applications in radiology has allowed for increased automation of radiological assessments^7^. This has included using DL for automated applications of various imaging modalities, such as X-ray mammography^8^, multiplanar computed tomography (CT) lung module detection^9^, and ultrasound (US) of the prostate^10^. In the field of OA MRI, important work has been done to automatically segment knee images^11^ and to detect lesion severity in cartilage, bone marrow, meniscus, and anterior cruciate ligaments (ACL)^12^. AI has also been demonstrated to detect meniscus tears and predict osteoporotic fractures, as well as to generate quantitative relaxation maps^13–15^. Additionally, several approaches have been suggested for reducing data acquisitions times using AI, including both approaches that construct images from undersampled k-space data as well as methods to improve image data^16,17^. Such developments have led to increased optimism of AI and DL improving the value of MRI as a high-end diagnostic modality by increasing its throughput and reducing its cost^18^.

**Figure 1:**
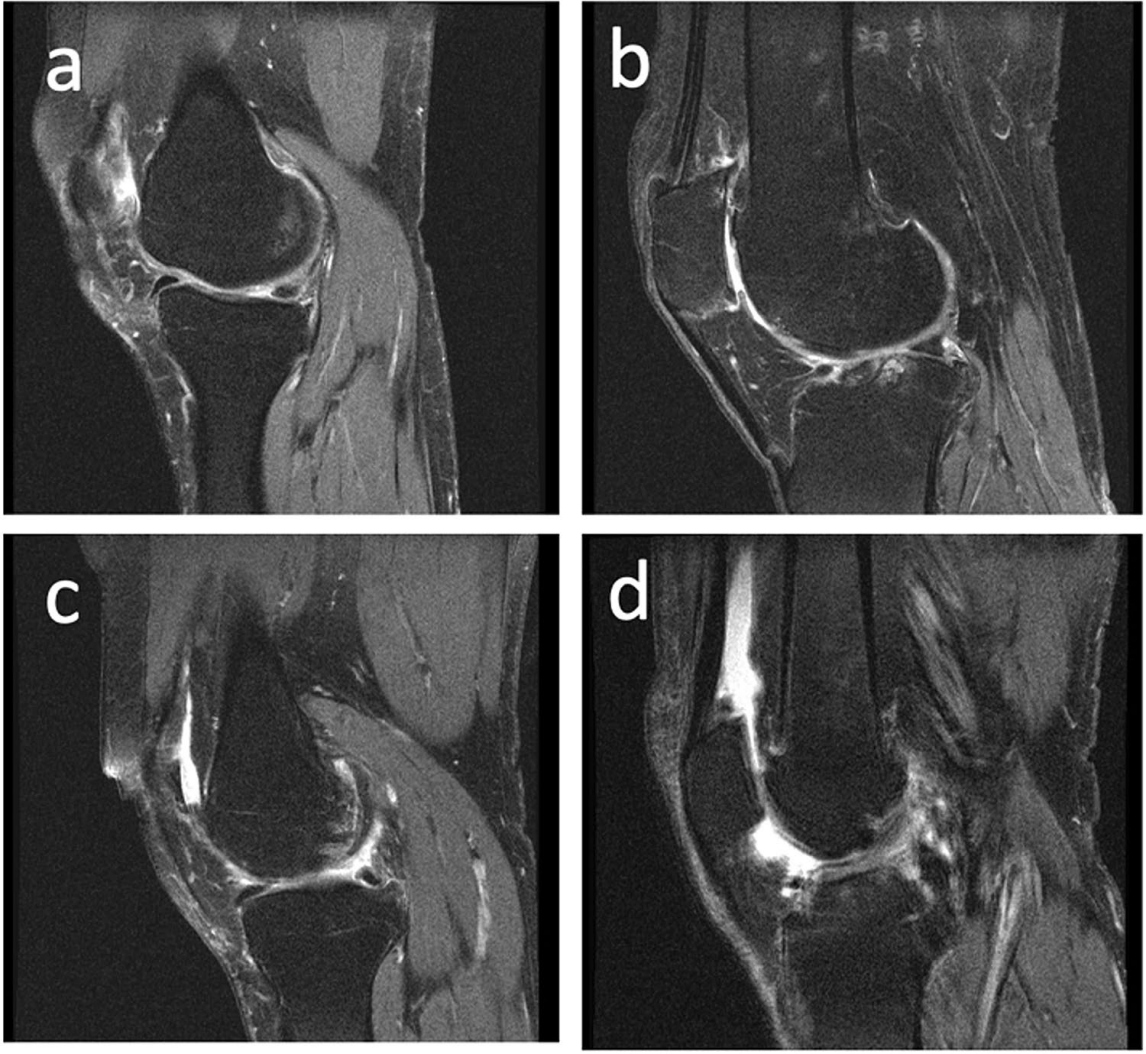
Examples of subjects with (**a**) Normal knee (MOAKS score 0), (**b**) Small effusion (MOAKS score 1), (**c**) Medium effusion (MOAKS score 2), and (**d**) Large (MOAKS score 3) effusion. In this work, a binary classification is used, with images as in panel (**a**) classified as without effusion, while images as in panels (**b**–**d**) are classified as with effusion.

Joint effusion, or accumulation of fluid, is commonly seen in OA patients^19^. This feature of OA has been associated with increased levels of pain^20^ and risk of cartilage loss^21^, demonstrating the importance of accurate assessment of effusion in OA patients. Although the composition of effusion is not well understood, its severity is correlated with the inflammation present and graded as mild, moderate, or severe. While there are multiple methodologies for quantitatively assessing effusion severity^19^, one commonly used metric is the MRI Osteoarthritis Knee Score (MOAKS) effusion-synovitis score^22^. This metric takes into account the fluid equivalent signal within the joint cavity on images with T2-, intermediate-, or proton-density-weighted contrast including synovitis and effusion and therefore uses the term effusion-synovitis, and will be referred to as MOAKS-ES in this work for brevity. This is scored on a scale of 0–3, with 0 being a physiologic amount, 1 being small (fluid continuous in the retropatellar space), 2 being medium (with slight convexity of the suprapatellar bursa), and 3 being large (with evidence of capsular distention). Examples of sagittal TSE images with each of these grades are shown in Fig. 1. This methodology has been used in several studies, including using data from the Osteoarthritis Initiative (OAI), a large multi-center study evaluating close to 5,000 OA patients^23,24^. However, this assessment often requires manual evaluation by a radiologist of a high-resolution image in the axial plane, which can become a laborious process for large patient volumes. Furthermore, such assessment can result in substantial inter- and intra-rater disagreements^22^. Additionally, several benefits would result from enabling effusion grading based on low-resolution images. A lower-resolution scan, employing fewer phase encodes, would shorten the scan, improving the patient experience and potentially increasing scanner throughput. Low-resolution processing also has value when using low-field MRI devices. This technique has seen substantial development in recent years and allows low-cost medical imaging in settings not applicable for conventional scanners, but typically acquires lower-resolution data^25–29^. There is therefore an unmet need for automating effusion estimation from MR images, including from images acquired non-axially with a low resolution.

In this proof-of-principle study, we examine the performance of a dense Neural Network (dNN) to automatically detect effusion from low-resolution sagittal Turbo Spin Echo (TSE) MR images and whether it can perform comparably to a human reader. We train the network on images from the OAI data set and associated MOAKS-ES labels provided with the publicly available OAI data. Images with MOAKS-ES value of 0 were separated from images of MOAKS-ES value of 1, 2, and 3, to enable a binary classification. The MOAKS-ES = 0 and MOAKS-ES > 0 will be referred to as datasets without effusion, or normal knees, and knees with effusion, respectively. We demonstrate that the dNN classifies effusion in a low-resolution test data set with 163 knees with a mean accuracy of about 62%. We compare the accuracy of the dNN to VGG16, a commonly used convolutional network. We also conduct a reader comparison, obtaining a binary effusion classification in a limited set of 36 patients both from our dNN as well as from an experienced musculoskeletal radiologist, and compare how well these agree with the classification labels included with the OAI, hereafter referred to as the ground truth classification labels. Such an approach could potentially allow automatic estimation of effusion from low-cost, low-field scanners or quick low-resolution images from standard clinical scanners, as well as possibly substantially reducing workloads for musculoskeletal radiologists assessing effusion severity.

## Materials and methods

The images for this study were collected as a part of the OAI between 2004 and 2006 using its first (baseline) timepoint. This involved imaging 4,796 male and female participants with, or at risk of, knee OA with Siemens Trio 3T MRI scanners, in addition to collecting other clinical and radiographic information^23,30^. Imaging was performed at 4 different imaging centers and data coordination performed at a fifth center. The participants provided informed consent as required by the Institutional Review Boards of the respective imaging centers (the Human Research Protection Programs or Offices of The Ohio State University, Columbus; University of Maryland School of Medicine, Baltimore; University of Pittsburgh School of Medicine; Brown University and Memorial Hospital of Rhode Island, Pawtucket), and the results were made publicly available for scientific investigation and OA drug development, with data coordinating centered at the University of California, San Francisco School of Medicine. We had institutional approval for use of this data. All methods were carried out in accordance with relevant guidelines and regulations.

### Data preprocessing

**Figure 2:**
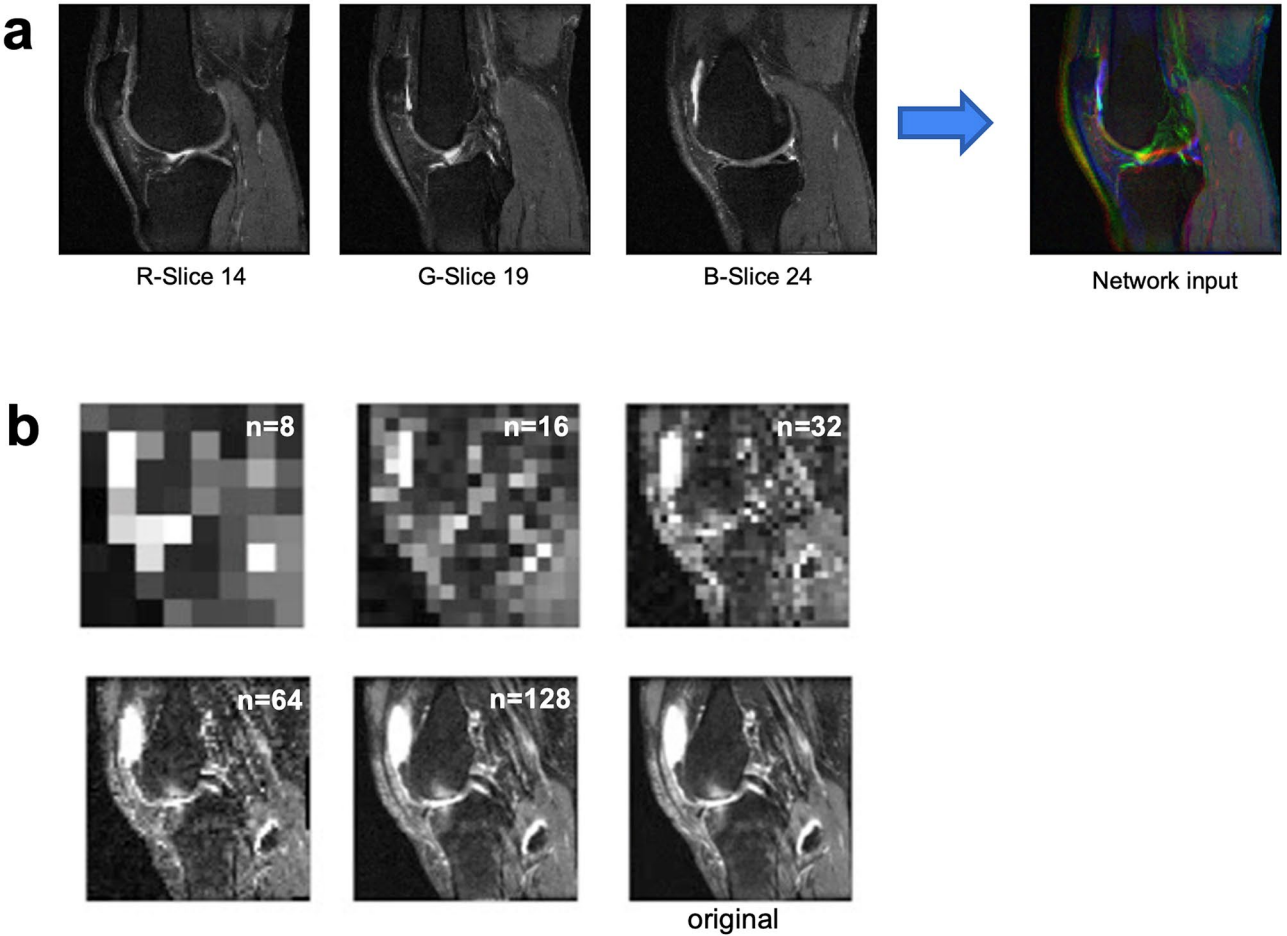
(**a**) Data creation through image slice combinations. Input images are created by treating intermediate slices as red, green, and blue color channels, and stacking them. Augmented images are created by stacking adjacent intermediate slices. (**b**) Example of image energy retained with progressively higher resolution.

The OAI obtained several different MRI pulse sequences, with the data stored as digital imaging and communication in medicine (DICOM) files. Of these sequences, this study used sagittal TSE images as the dNN input to predict the effusion class, due to it giving the highest fluid contrast of the sequences available. The sequence parameters are shown in Table 1. While the TSE scans typically contained 37 slices as shown in Table 1, our approach was to format the input data in the form of 2D color images, both to enable comparisons with image-based networks such as VGG16 as well as to reduce the amount of input data used. To achieve this, we selected three uniformly distributed slices (14, 19, and 24). We then combined these into a single color image by associating the slices in ascending order with red (R), green (G), and blue (B) channels respectively, as shown in Fig. 2a. In addition, the DICOM images had been initially constructed at a resolution of 448 × 448 pixels. As one of the goals of this study was to investigate performance with limited data, this image resolution was lowered. This was done by inspecting several image sizes and evaluating the amount of energy retained, defined as its mean square intensity; the energy was then compared to the energy of the original image with the goal of retaining at least 99% of the energy. Images resized to n × n pixels, with an n of 2, 4, 8, 16, and 32, didn't retain comparable energy as the original scan (> 1% difference). Conversely images with n of 64 and above closely matched the original scan (< 1% difference), as shown in Fig. 2b. Based on this analysis, all scans were resized to 128 × 128 pixels using Python’s (version 7.2.2) cv2 library. This resulted in an in-plane resolution of 1.25 mm, similar to what is reported in recent low-field studies.

**Table 1:** Scan parameters for the sagittal TSE sequence used for effusion prediction.

Plane	Sagittal
Matrix (frequency × phase)	448 × 313
Slices	37
Field of View (mm)	160
Slice thickness/gap	3/0
Flip angle (°)	180
TR/TE (ms/ms)	3200/30
Bandwidth (Hz/pixel)	248
Averages	1
Echo train length	5
Phase encode axis	A/P
Scan time (min)	4.7

Patients with incomplete imaging or without effusion labels were excluded from the study. After discarding incomplete radiological data, there were 1628 total scans, including 882 left knees and 746 right knees. The characteristics of the data set, including female/male ratios and mean age, effusion grade, and the commonly used OA severity measure of Kellgren Lawrence (KL) grade^31^ are shown in Table 2.

**Table 2:** Patient characteristics of data set.

% female	61.8
% male	38.2
Mean age	61.7
Effusion grade % (0, 1, 2, 3)	43.5/37.2/14.4/4.8
Mean KL grade (right knee)	1.01
Mean KL grade (left knee)	1.04

To ensure adequate testing of our method, 10% of the original data (163 scans) was set aside. Of this testing data, 36 scans were utilized for performance comparison between the trained network and a musculoskeletal radiologist. This test data subset contained an equal number of scans from both left and right knees, as well as 18, 6, 6, and 6 scans for MOAKS-ES values of 0, 1, 2, and 3 respectively.

**Figure 3:**
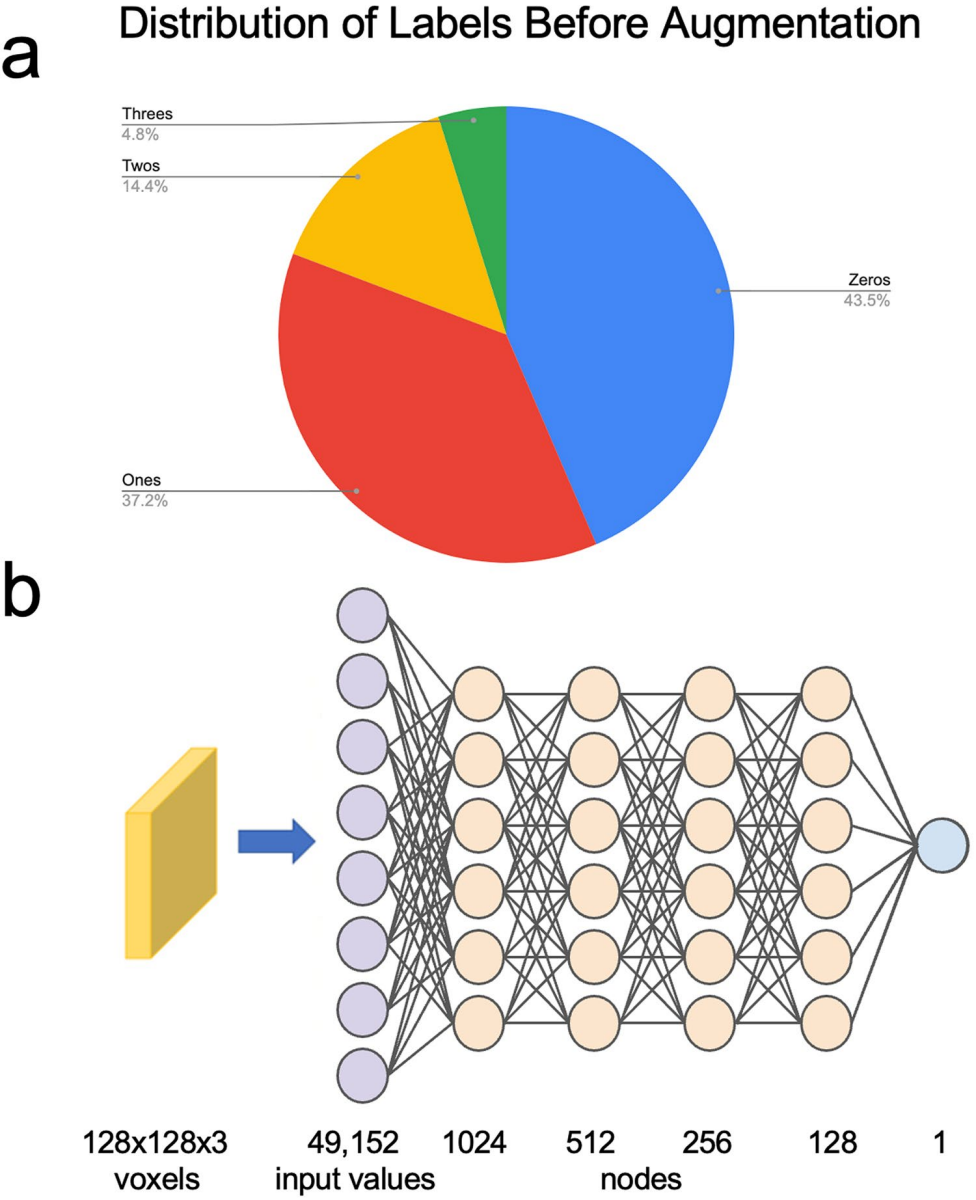
(**a**) Initial distribution of MOAKS-ES labels before data augmentation. (**b**) Schematic of network architecture. A flattened 49,152-valued input layer is followed by dense layers with 1,024, 512, 256, and 128 nodes, with 10% dropout after the first dense layer.

The rest of the data was non-uniformly distributed with 633, 542, 214, and 76 scans having effusion values of 0, 1, 2, and 3, respectively. Optimizing on such non-uniform data resulted in predicting no effusion (MOAKS-ES value of 0) or effusion (MOAKS-ES values of 1, 2, or 3) for all scans. To obtain uniformly distributed data with equivalent representative images for normal and higher effusion levels, we performed data augmentation on images of effusion value 0, 2, and 3, and utilized different slice combinations to create new color images. This was achieved by taking the slices adjacent to the original and combining to create a single, color image. For example, the original data was constructed using slices 14, 19, and 24 while augmented data was assigned the same label, and constructed using slices 14 + n, 19 + n, 24 + n where n ranged from -3 to 4 and was not equal to 0. After augmentation the data was equally balanced with MOAKS-ES values of 1, 2, and 3 having 542 representative scans each, and MOAKS-ES value of 0 having 1626 representative scans. The original distribution of the labels before augmentation is shown in Fig. 3a.

The split validation method was utilized by randomly splitting the remaining data with the proportions of 80% and 20% (2599 and 653 scans) into the training and validation subsets respectively. The data distribution was checked to assure equal distribution between knees with and without effusion. Since the testing subset was separated prior to augmentation, no additional data was added, to ensure no data leakage from augmentation.

### Network design

Currently, pre-trained models are widely used for image classification problems as they’ve already been trained on millions of images. These networks are considered state-of-the-art (SOTA) and are quite large with billions of parameters, allowing the network to achieve relatively high levels of accuracy. In this work, however, we chose a feed-forward neural network with relatively few parameters as it would allow us to have complete control over its structure and parameters and be less computationally intensive. Its performance was then compared to VGG16, a popular SOTA network^32^. In its final design, the architecture was a fully connected five-layer neural network with a 10% dropout after the first dense layer. After flattening the 128 × 128 × 3-sized image data into a 49,152-valued input, the data was passed through successive dense layers with 1,024, 512, 256, and 128 nodes, as shown in Fig. 3b. The final classification layer consists of one node and uses the sigmoid activation function for categorizing the MR images into the two labels: 0 representing physiologic fluid levels (MOAKS-ES value of 0), and 1 representing higher fluid levels (MOAKS-ES values of 1, 2, or 3). For both VGG16 and the dNN, we optimized the hyper-parameters including learning rate, batch size, epochs, and activation function. Training, validation, and testing results were compared between the two networks.

The dNN was trained using the Adam optimizer on a binary cross-entropy loss function, a batch size of 300, and learning rate of 0.0004. The network was set to train for a maximum of 50 epochs, with early stopping applied, so that if 4 epochs passed without improvement in the validation loss, the training stopped. The weights of VGG16’s final layer, with other layers pre-trained using ImageNet, were trained using the RMS-prop optimizer on a binary cross-entropy loss function with a batch size of 20 and learning rate of 0.0001, using the same early stopping condition. The network was set to train for a maximum of 50 epochs. Both networks were compiled using Tensorflow (v. 1.13.1) with Keras (v. 1.0.8) backend on Python (v. 3.7.1). The networks were trained on the training subset and hyperparameters tuned with the validation subset. Development took place on a Dell Latitude 7390 computer with Intel i5-8350U CPU, and a 16.0 GB RAM.

After testing the dNN, robustness to reduced SNR was evaluated by adding zero-mean Gaussian noise to all test images with a standard deviation of 5%, 10%, 15%, 20%, 25%, and 30% of the maximum test data intensity. Accuracy was determined by the percentage of categorizations where the predicted class matched the ground truth class. ROC curves, associated AUC values, and Matthews Correlation Coefficient (MCC) calculations were implemented to analyze the dNN’s performance for no added noise and 5–30% added noise. Robustness was assessed to account for the high variability in biomedical images and gauge realistic applicability. To assess the regional sensitivity of the network, an activity map analysis was performed. As the network was not convolutional, this was done using occlusion sensitivity computation^33^ instead of techniques such as grad-CAM^34^.

### Reader comparison

As previously mentioned, a smaller test set of 36 test subjects, included in the 163-subject test set, was used for reader comparison. No augmentation was performed on this data. The data was distributed with 18, 6, 6, and 6 scans representative of effusion values 0, 1, 2, and 3 respectively. For each effusion value, half the scans were from the left knee and half were from the right knee. A musculoskeletal radiologist reader with 28 years of experience evaluated images from these subjects with the same 128 × 128 × 3 resolution as used with the dNN, and assigned them an estimated binary class. The agreement between the reader and the ground truth class, based on MOAKS-ES scores, was examined by calculating the percentage of subjects where the reader agreed with the ground truth class as well as calculating the mean error and mean absolute error, with error defined as the ground truth class subtracted from the reader class. The same analysis was then performed for the classes predicted by the dNN. The mean errors of the two approaches were statistically compared by applying a two-tailed t-test with α = 0.05. The agreement between the reader assessment and dNN assessment was evaluated with a Cohen’s kappa calculation.

## Results

**Figure 4:**
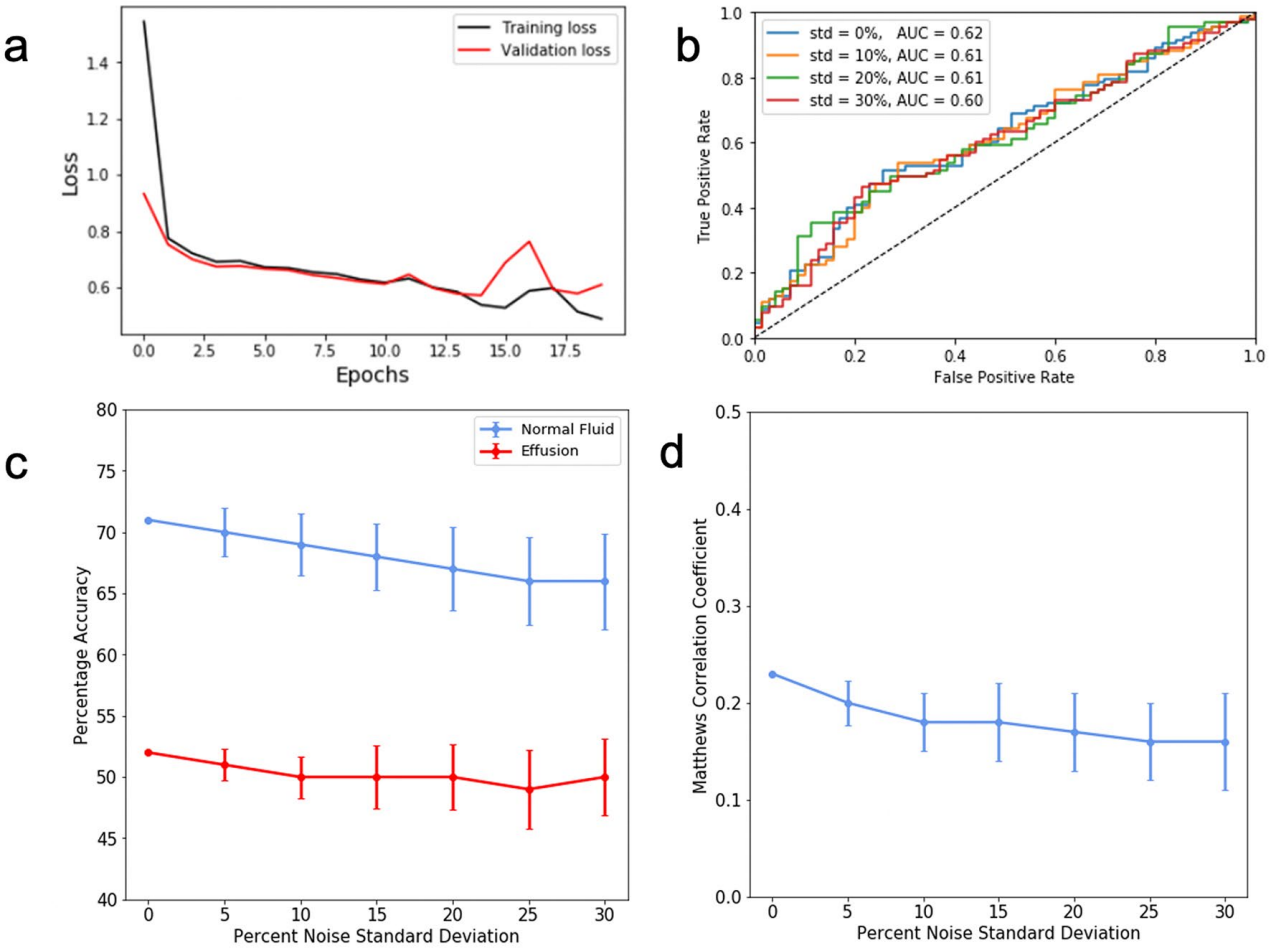
(**a**) Learning curves for the training and validation data sets of the dNN. (**b**) The testing data was degraded with additive Gaussian noise with a standard deviation varying from 0 to 30% of the maximum pixel intensity. ROC curves were recorded, and the AUC was calculated. (**c**) The same method as panel b, mean and standard deviation of accuracy was recorded for each class. (**d**) The same noise addition method as panel b, mean and standard deviation of Matthews correlation coefficient was recorded with additive noise.

**Figure 5:**
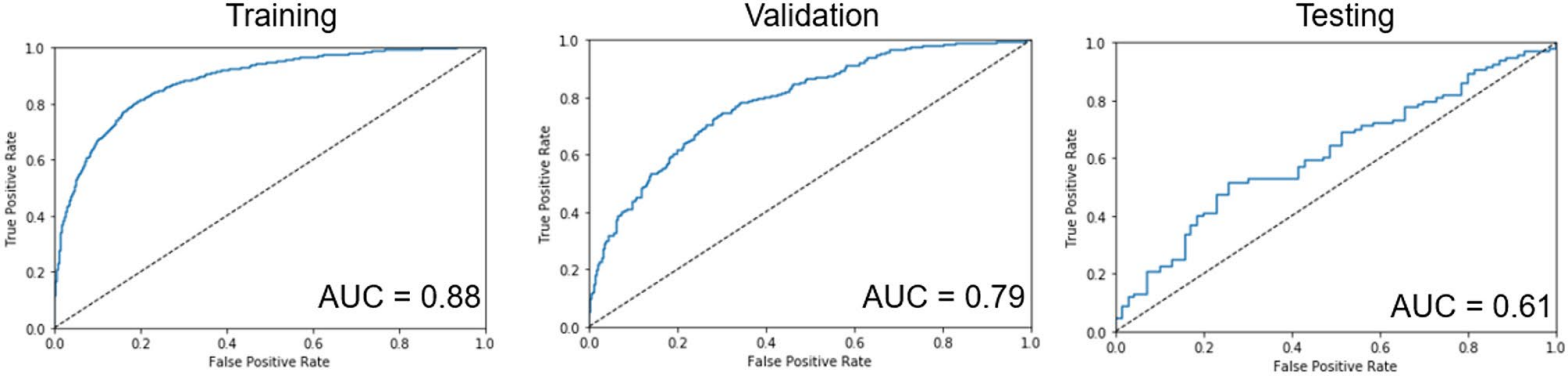
The dNN’s training, validation, and testing ROC curves with associated AUC values.

The training of the dNN took about 9 s per epoch, resulting in a total training time of approximately 3 min. The training stopped due to the validation loss curve flattening after 20 epochs, as shown in Fig. 4a. For the test data set, the dNN achieved a 71% and 52% accuracy for classifying images without and with effusion, respectively, giving an average accuracy of 61.5%. Figure 4b shows the minimal change to the area under the testing ROC curve (AUC) with additive noise. Similarly, accuracy degradation to increasing percentages of noise is shown in Fig. 4c. The figure shows that even for very strong additive noise levels, up to 30% of the maximum signal intensity, the accuracy of the network remained good. Figure 4d demonstrates the minimal degradation to MCC, which measures the quality of binary classifications on a scale from − 1 to 1, even with increasing noise. ROC curves and associated AUC values for training, validation, and testing subsets are shown in Fig. 5.

The training of VGG16 took about 72 s per epoch, resulting in a total training time of approximately 1 h. The training stopped when the validation loss curve did not appreciably decrease with time. For the larger test data set, VGG16 classified all images into the effusion class, giving an average accuracy of 50%. Despite multiple attempts at hyper-parameter tuning, all results yielded an equivalent or even lower agreement. Therefore, the AUC for testing data was consistently less than or equal to 0.5.

Table 3 shows the results from the comparison of the reader assessments to the dNN assessments for the smaller 36-knee test data set. On average, the dNN had a 47.2% agreement with the OAI scores, while the reader had a 41.7% agreement. The dNN had a higher agreement than the reader for knees without effusion, while having slightly lower agreement for the knees with effusion. The mean error of the dNN was − 0.19 and for the reader it was − 0.08. This difference was not determined to be statistically significant based on the described *t*-test (*p* > 0.05). The mean absolute error for the dNN was 0.53, while for the reader it was 0.58. Calculation of Cohen’s kappa between the reader and the dNN resulted in an index of 0.29.

**Table 3:** Comparison of reader network label estimates for a 36-subject data set. “Correct” is defined as giving the same effusion estimate as given in the OAI. “Error” is defined as the OAI label subtracted from the estimated label.

	Total correct (%)	No effusion correct (%)	Effusion correct (%)	Mean error	Mean absolute error
Reader	41.7	50	33.3	− 0.08	0.58
Network	47.2	66.7	27.8	− 0.19	0.53

**Figure 6:**
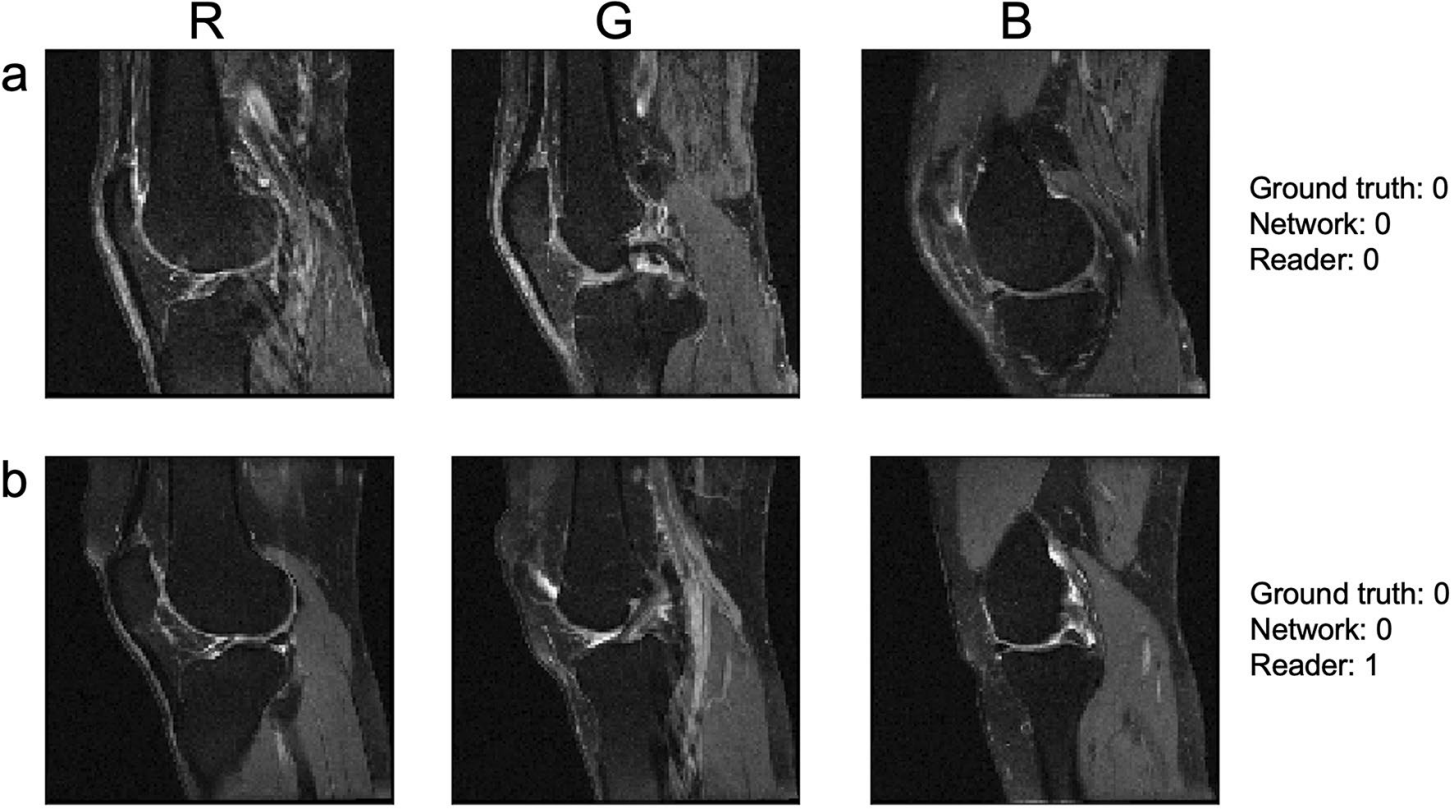
Two sample subjects, one with agreement between the dNN and the reader and the other with disagreement. As described in Fig. 2, the three slices were combined into a color image as shown in the top labels to facilitate classification by 2D image-based networks. (**a**) A subject where the dNN and reader assessments agreed with the OAI class (both giving a value of 0). (**b**) A subject where the dNN agreed with the ground truth of normal fluid amounts, while the reader estimated effusion.

Figure 6 shows examples of where the reader and the dNN disagreed. In Fig. 6a, the dNN and the reader both agreed with the ground truth value of normal fluid amount, while in Fig. 6b the dNN agreed with the ground truth of normal fluid while the reader assessed the data set to represent a knee with effusion (MOAKS-ES > 0).

**Figure 7:**
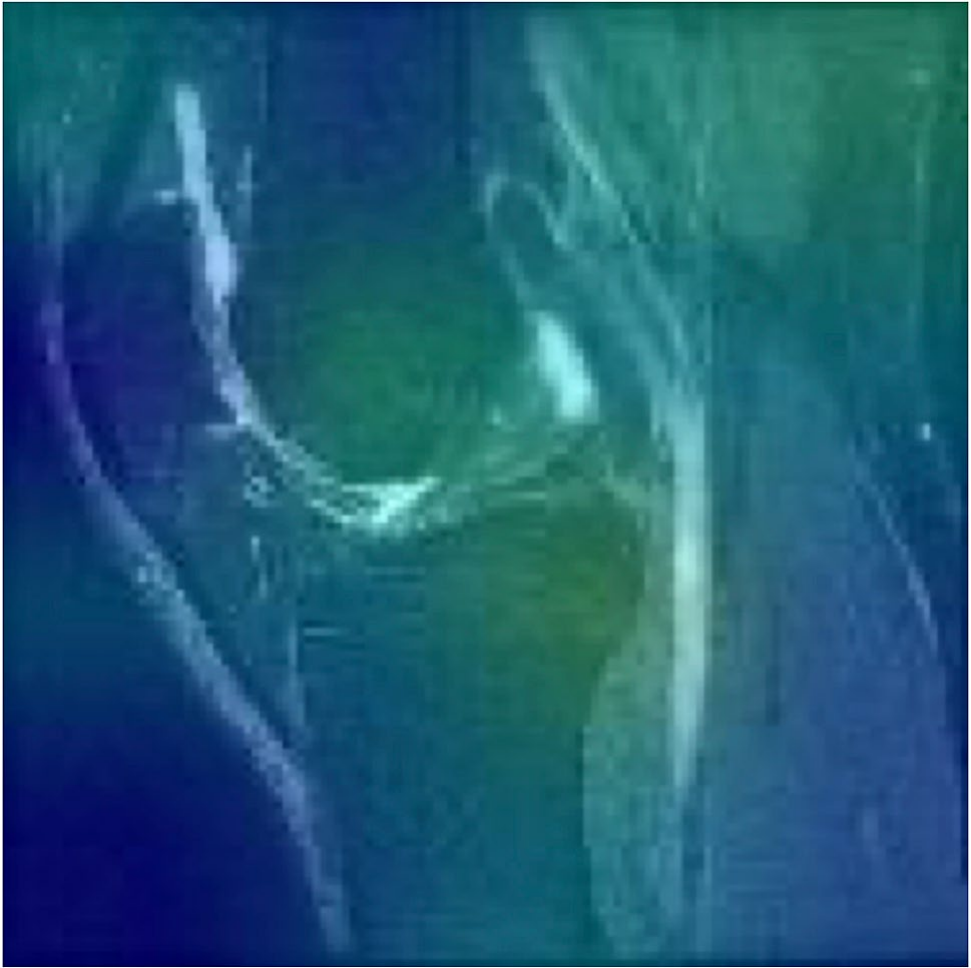
Sample occlusion map, demonstrating regions of high sensitivity for classification.

A sample occlusion map, demonstrating which regions the network was sensitive to when making a classification, is shown in Fig. 7. The map shows increased sensitivity in the joint space and around the suprapatellar bursa, although some variability in such maps was noted.

## Discussion

In this study we investigated, as a proof-of-principle, the feasibility of a deep learning system playing a role in classifying low-resolution MRI images into categories of no effusion (physiological fluid amount, MOAKS-ES = 0) or having effusion (MOAKS-ES > 0) with a comparable accuracy as a human reader, which has relevance for low-cost MRI. The performance of our proposed model was trained and evaluated on images from the Osteoarthritis Initiative (OAI) and MOAKS-ES labels available with that public data set. Performance analysis demonstrated that this methodology holds promise, performing with comparable or better accuracy to a radiologist when using low-resolution data.

As one of the goals of automatic effusion classification as presented in this work is to reduce radiologist workload, the classification accuracy should ideally be similar to reported inter-rater agreement for effusion grading. A previous study analyzed the reliability of reading MRI Osteoarthritis Knee Scores (MOAKS) features including effusion-synovitis values^22^. In that study, two expert radiologists with 8 and 10 years of experience assessed MRIs of 20 knees from the OAI, resulting in an inter-rater percent agreement of 0.70 when assigning effusion values. In another study, where two trained orthopedic surgeons evaluated 160 knees using MOAKS, interclass reliability was reported as 0.72^35^. Although our model only achieved 61.5% binary classification accuracy on average for the larger test data set, given the inherent subjectivity of effusion estimation and the fact that our data was much more limited in terms of resolution and slice range, we consider the results promising.

As already described, we applied data augmentation in this work to get a more even distribution of MOAKS-ES training labels as well as to generally have more data for training. In machine learning, such augmentation is often achieved by operations such as flipping, rotating, or cropping the original images. We did not see this as a realistic approach in this work, as the field of view and patient orientation are quite standardized during the scan by a professional MRI technologist. We therefore used the approach of generating new slices by combining adjacent original slices as described, maintaining the patient orientation and field of view.

The testing results demonstrate that the dNN performs substantially better than the VGG16 network in distinguishing normal from high fluid levels. The area under the VGG16 ROC test curve was 0.5, indicating that the VGG16 approach is incapable of distinguishing between the two classes and only classifies with an accuracy equal to random chance. Since the classification between normal fluid levels and effusion is often subtle, perhaps the data, even after augmentation, is not enough to fully train the multi-million parameters. The superior performance of the dense network could also indicate the importance of localized pattern detection in the image instead of globally detected patterns.

It should be kept in mind that complete agreement of the dNN with the ground truth effusion classification, while ideal, is not the primary goal of this project, but rather to see whether it achieves equivalent or better agreement than a human reader. The reader comparison of 36 subjects in the smaller test data set suggests that a dNN can perform as well or better than a trained musculoskeletal radiologist in assessing binary effusion classification, based on MOAKS-ES scores, using low-resolution sagittal TSE images. The rate of agreement between the reader and the dNN was observed to be 67%. Calculation of Cohen’s kappa gave the probability of random agreement between reader and network in the 36 images as 0.53, suggesting that agreement with the reader was more than random, and yielded an index of 0.29, indicating fair to good agreement beyond random chance. The mean absolute difference between reader and dNN classification was 0.33. Notably, the network performed better than the reader for images without effusion and comparably for images with effusion. The results could indicate that using neural nets for low-resolution effusion scoring might help when making a binary choice between normal knees and knees with effusion, but it should be kept in mind that this 36-knee test data set is quite small and a larger study would need to be performed to assess this with statistical significance. With only 18 data sets per class (with one misclassified point resulting in a 5.6% error for that class), some statistical variability is to be expected, which was indeed observed even without change in hyperparameters. A larger data set, while more demanding of the reader, could reduce such variability. Nonetheless, the result from even this small data set serves as an indication that matching or outperforming a human reader is feasible.

An important aspect of this work is the capability of the dNN to detect effusion from low-resolution 128 × 128 images. As MRI scan time increases with the number of phase encodes, one could reduce the scan time of the original 448 × 313 high-resolution acquisition by approximately a factor of 2.5. Given that our network performed well compared to a radiologist with this resolution, the dNN could help to accurately diagnose patients with a substantially reduced MRI scan time. This is beneficial to the patient, as long scan time can lead to patient discomfort^36^ and potential motion^37^, leading to image artifacts. This could be especially relevant for OA patients due to the pain commonly associated with OA^38^. Furthermore, shorter scan time can lead to higher patient throughput, and thus potentially reducing the economic burden of OA diagnosis and monitoring^39^ as well as improving patient experience and clinical effectiveness^40,41^. Some scanner systems, including low-cost, low-field portable MRI scanners, use lower resolution due to system limitations^26,28^. This study demonstrates that data from such scanners could possibly be automatically classified for effusion, potentially helping to expand the possible user base for such systems, allowing more widespread use of medical imaging. Additionally, the lower resolution results in reduced data size, requiring less memory for post-processing, and the ability to process lower-resolution data also allows for improving the signal-to-noise ratio (SNR) by using larger voxels.

Although the presented model showed promise, it has some limitations. Firstly, effusion classifications are inherently subject to interpretation, leading to reader disagreements and variability in any effusion assessment, including the ground truth scores. Second, our data set was deliberately kept limited, both in terms of resolution and slice range, to emulate the conditions of technically limited scanner systems and to enable comparisons with 2D image-based networks. This can result in certain anatomical details, relevant for MOAKS-ES classification as described in the Introduction, to be missing from the data. The method attempts to predict the effusion classification in spite of such limitations, but this will undoubtedly lead to less accuracy than for data sets with higher detail. Using other effusion metrics, such as volumetric quantification of fluid^42,43^, might yield different levels of agreement. Additionally, as has already been described, the small size of the 36-knee data set inevitably results in variability and makes statistical comparisons challenging, and a larger data set for reader comparisons would likely be beneficial. Finally, the available OAI data contained relatively few radiological scans with a MOAKS-ES value of 2 and 3. As a result, the model in the paper has not been trained with high variability in this category and is potentially not as robust at classifying images with very high fluid levels. Training the network further and refining parameters when more data is available would be a valuable future direction of this work.

## Conclusion

In this proof-of-principle work, we estimated the feasibility of classifying knee effusion using neural networks. We demonstrated that having a neural network classify low-resolution images into categories of effusion or no effusion with an accuracy comparable to a radiologist is feasible. This has relevance for low-cost, low-resolution knee scanning and could also be integrated into clinical osteoarthritis studies to save scan time and reduce radiological work.
